# Surgical Versus Non-surgical Treatment of Unstable Lateral Compression Type I (LC1) Injuries of the Pelvis With Complete Sacral Fractures in Non-fragility Fracture Patients: A Systematic Review

**DOI:** 10.7759/cureus.29239

**Published:** 2022-09-16

**Authors:** Jonny R Varma, Michael Foxall-Smith, Richard L Donovan, Michael R Whitehouse, Chris Rogers, Mehool Acharya

**Affiliations:** 1 Trauma and Orthopaedics, North Bristol NHS Trust, Bristol, GBR; 2 University of Bristol, Musculoskeletal Research Unit, Bristol, GBR; 3 Musculoskeletal Research Unit, University of Bristol, Bristol, GBR

**Keywords:** screw fixation, pelvic surgery, pelvic ring, pelvic ring injury, closed pelvic fracture

## Abstract

Lateral compression type 1 (LC1) injuries comprise two-thirds of pelvic fractures. Approximately one-third of LC1 fractures are unstable and may benefit from surgical fixation to improve stability but it is not clear if this leads to better clinical or cost-effectiveness outcomes. This study explores differences in patient-reported outcomes, complications, time-to-mobilisation, cost-effectiveness, and length of hospital stay between surgically and non-surgically treated unstable LC1 non-fragility fractures.

We performed a systematic review to determine whether surgical or non-surgical treatment yielded better clinical and cost-effectiveness outcomes for the treatment of unstable LC1 pelvic injuries with complete sacral fractures, excluding fragility fractures. We searched Medline, Embase and Cochrane databases from inception to June 2022, as well as clinical trial registries. A formal meta-analysis was not possible due to available study designs and heterogeneity. Therefore, a narrative review of the findings has been provided.

Five observational studies met the inclusion criteria. A total of 183 patients were treated surgically, and 314 patients were treated non-surgically. Patients treated surgically had lower pain levels (Visual Analogue Scale) and fewer days to mobilisation. Quality of life (EuroQol-5 domains and 36-Item Short Form questionnaires) was better in the surgical group, but not statistically significant. No statistical differences in the length of hospital stay or complication rates were found.

This review highlights the low quantity and quality of existing data on patients with unstable LC1 pelvic fractures and the need for a definitive randomised controlled trial to determine whether surgical or non-surgical care should be the preferred treatment concerning clinical and cost-effective care.

## Introduction and background

Lateral compression (LC) fractures are the most common type of pelvic fracture, with lateral compression type 1 (LC1) injuries accounting for two-thirds of all pelvic fractures [1,2]. Unstable LC1 fractures are synonymous with the 61B2.1 pelvic ring fractures of the Orthopaedic Trauma Association (OTA)/AO Foundation (AO) classification [3]. LC1 fractures typically result from a lateral force, which frequently leads to pubic rami fractures of the anterior pelvic ring and an associated sacral impaction fracture [4]. The mortality rate of LC1 fractures during index hospital admission is reported to be between 5% and 9% [2].

LC1 fractures may be stable or unstable, which has caused controversy over the optimal management strategy for this injury pattern [5-7]. There is currently no high-quality evidence or agreed surgical strategy for their management. Sagi et al. reported that up to 37% of LC1 fractures are unstable, highlighting the potential role of surgical stabilisation [8]. Surgical fixation may improve time to mobilisation, reduce complications associated with prolonged immobility, and improve long-term functional outcomes [9-11]. Despite this, the risk of surgical site infection, neurovascular injury, reoperation, and the risks of general anaesthesia are potential risks of surgical intervention [10].

Historically, LC1 injuries have been managed non-surgically with restricted weight-bearing, physiotherapy, and walking aids [4]. The contemporary rationale for surgical management of LC1 injuries is to achieve stability, but this depends on the independent clinical judgment of the stability of the injury, which has been demonstrated to be inconsistent among surgeons [9,12]. Uncertainty concerning the definition of ‘instability’ remains; some surgeons state it depends on the tendency of the fracture to displace over time, while others define it in terms of excessive pain on mobilisation regardless of the bone position (adopting the definition of ‘functional’ instability) [6,12].

This study aims to systematically review the existing literature to explore if there are any differences in patient-reported outcomes, complications, time-to-mobilisation, cost-effectiveness, and length of hospital stay between surgically and non-surgically treated unstable LC1 non-fragility fractures.

## Review

Methodology

Data Sources and Search Strategy

We performed a systematic review per the Preferred Reporting Items for Systematic Review and Meta-Analysis (PRISMA) guidelines based on a pre-defined methodology registered with the International Prospective Register of Systematic Reviews (PROSPERO ID: CRD42021256075). We systematically searched Medline, Embase and Cochrane (Central) databases from inception to 5 June 2022 for studies reporting on patient-reported outcome measures following surgical versus non-surgical management of unstable LC1 pelvic fractures. The International Clinical Trial Registry Platform was also searched for completed, ongoing, and registered trials. The search terms included a combination of Medical Subject Heading (MeSH) terms, synonyms, and related terms for LC fracture, surgical fixation, and non-surgical management. The detailed search strategies are outlined in Appendix 1. The results of the searches were then integrated into Rayyan [13] online bibliographic software.

One reviewer (JV) reviewed the titles and abstracts of retrieved studies for inclusion suitability and then obtained the full texts of potentially relevant articles for further evaluation. Two reviewers (JV, MFS) independently evaluated the full texts of the articles identified, and any disputes regarding eligibility criteria were discussed with and resolved by a third author (RLD).

Eligibility Criteria and Data Extraction

Randomised controlled trials (RCTs) and observational studies were eligible for inclusion. The primary outcome was patient-reported outcome measures (PROMs) such as the Iowa Pelvic Score, 36-Item Short Form Health Survey (SF-36), Oxford Hip Score, EuroQol-5 domains-3 level (EQ-5D-5L), and Majeed Pelvic Score; and patient-reported pain (Visual Analogue Scale (VAS), Brief Pain Inventory). Secondary outcomes included complications (wound complications, infections, neurovascular injuries, venous thromboembolic (VTE) events, such as deep vein thrombosis (DVT) and pulmonary embolism, chest infection, metalwork failure, non-union, malunion); re-hospitalisation; mortality; cost-effectiveness; and length of hospital stay.

Studies that (i) included patients with fragility fractures resulting from low-energy trauma (fall from standing height or less); (ii) included children aged 16 years or younger; or (iii) included animals or cadavers were excluded. Two authors (JV, MFS) independently extracted the relevant data from eligible studies. Any disputes were explored and resolved by a third author (RLD).

Risk of Bias and Data Synthesis

The Cochrane Risk of Bias in Non-randomised Studies - of Interventions (ROBINS-I) tool [14], a validated tool for assessing the quality of non-randomised studies, was selected to assess the risk of bias in observational studies. Continuous variables were represented as mean differences (MDs) or standardised mean differences (SMDs) with 95% confidence intervals (CIs). Risk estimates (risk ratios) were used as the standard measure of association across studies for dichotomous variables with 95% CIs. A two-sided statistical significance of 0.05 was used. Due to a paucity of data, we were unable to conduct a formal meta-analysis and have, therefore, provided a narrative review of the findings.

Results

Study Identification

**Figure 1:**
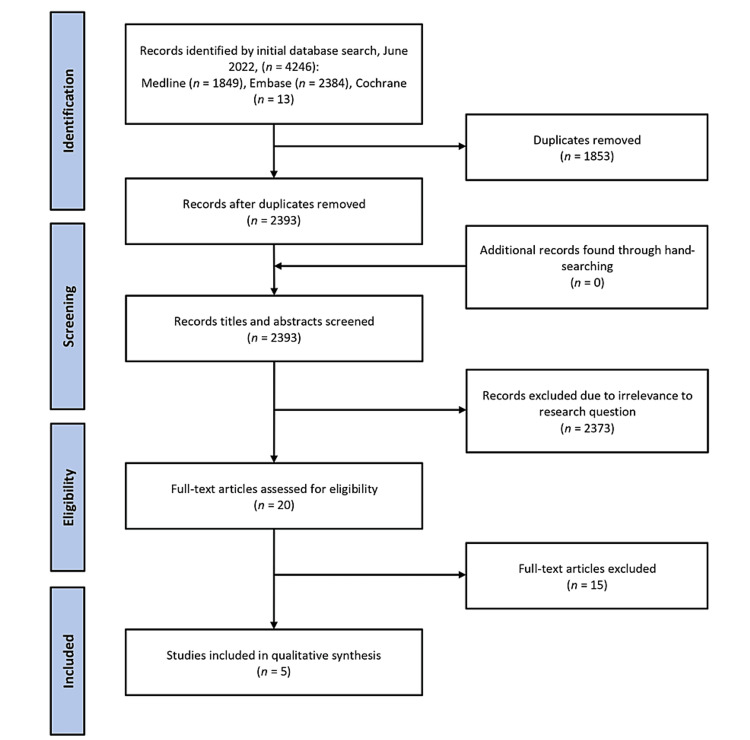
PRISMA flowchart. PRISMA: Preferred Reporting Items for Systematic Review and Meta-Analysis

The PRISMA flowchart in Figure 1 summarises the study identification and selection process in detail. After removing 1,853 duplicates from the initial searches, 2,393 studies were screened; 2,373 studies were removed after screening titles and abstracts, and an additional 15 studies were excluded after full-text evaluation. Finally, five studies [15-19] were included in this systematic review.

Study Characteristics

Table 1 summarises the characteristics of the studies included in the review. The included papers were published between 2016 and 2021. A total of 490 LC1 (or equivalent) injuries were included, of which 183 (37%) were treated surgically. Four studies were retrospective observational studies [15-18], and one was a prospective study [19]. All studies compared surgical versus non-surgical treatment of LC1 (or equivalent) injuries. Most patients were female and in their third or fourth decades of life. Two studies were performed in the United States and three in Europe (Germany and Lithuania). Three studies used shorter-term follow-up time points ranging from 24-48 hours [15] to 1-12 weeks [16,19] post-injury, one study had a mean follow-up period of 47 months (standard deviation (SD): 13-82 months) [17], and the largest follow-up duration was a mean of 52 months (SD: 48-84 months) [18]. Primary outcome data from the five individual papers are listed in Table 2.

**Table 1:** Characteristics of the included studies. LC1: lateral compression 1; SD: standard deviation

Source and study design	Participants and characteristics	Fracture classification	Intervention and comparator	Length of follow-up	Fixation technique
Hagen et al. (2016), United States. Retrospective observational study [15]	Age - Surgical: Mean = 35 (SD = 13.4). Non-Surgical: Mean = 41 (SD = 21); Sex - Surgical: 21 male; 30 female. Non-surgical: 53 male; 54 female	Sample size Young & Burgess - LC1 = 158	Surgical - n = 51. Non-surgical - n = 107	Post-injury - Time points at 24 hours and 48 hours after admission, and 24 hours before discharge, respectively; Time to Mobilisation - Timepoint at which patient achieved independent transfer from the bed to the chair	Posterior percutaneous fixation with or without anterior fixation (n = 51)
Höch et al. (2018), Germany. Retrospective observational study [17]	Age - Surgical: Mean = 39 (SD = 13.2). Non-Surgical: Mean = 35.6 (SD = 14.7); Sex - Surgical: 19 male; 17 female. Non-Surgical: 19 male; 16 female	Sample size OTA/Tile - Type B2.1 = 71	Surgical - n = 36; Non-surgical - n = 35	Post-injury - At least at 1 year, mean 47 months	Isolated percutaneous sacroiliac screw fixation (n = 25), isolated stabilisation of the anterior pelvic ring using plate osteosynthesis (n = 8), isolated percutaneous anterior screw fixation of the pubic rami (n = 1) with sacroiliac screw fixation (n = 1), external fixation only (n = 1)
Petryla et al. (2021), Lithuania. Retrospective observational study [16]	Age - Surgical: Mean = 35.84 (SD = 12.22). Non-Surgical: Mean = 40.11 (SD = 16.56); Sex - Surgical: 10 male; 27 female. Non-Surgical: 3 male; 15 female	Sample size OTA/Tile - Type B2.1 = 55	Surgical - n = 37; Non-surgical - n = 18	Post-injury - Timepoint I - before injury (pre-traumatic condition). Timepoint II - 10 weeks following injury	Anterior and posterior pelvic ring fixation (n = 23), posterior ring fixation only (n = 13), external fixation only (n = 1)
Tornetta et al. (2019), United States. Prospective observational study [19]	Age - Surgical: Mean = 40.1. Non-Surgical: Mean = 38.2; Sex - Surgical: 18 male; 26 female. Non-surgical: 53 male; 84 female	Sample size Young & Burgess - LC1 = 187, Combined = 1, Unknown = 6	Surgical - n = 50; Non-surgical – n = 144	Post-injury - Timepoints: 24 hours, 1 week, 3 weeks, 6 weeks, 12 weeks	Anterior and posterior fixation (n = 29), posterior fixation only (n = 19), anterior fixation only (n = 2)
Zwingmann et al. (2019), Germany. Retrospective observational study [18]	Age - Surgical: Mean = 43 (SD = 22.5). Non-surgical: Mean = 45.5 (SD = 25.1); Sex - Surgical: 2 male; 7 female, Non-surgical: 5 male; 5 female	Sample size OTA/Tile - Type B2 = 19	Surgical - n = 9; Non-surgical – n = 10	Post Injury - Mean 4.3 years (range = 2–7; SD = 2)	Sacroiliac screw fixation (n = 9)

**Table 2:** Summary of primary outcome data. CI: confidence interval; EQ-5D: EuroQol-5D Score; MCS: mental component score; PROMs: patient-reported outcome measures; PCS: physical component score; SF-36: 36-Item Short Form Survey; SD: standard deviation; VAS: Visual Analogue Score

Study	PROMs	Pain	Other secondary outcomes	Length of hospital stay
Hagen et al. [15]	Not measured	10-point VAS - Change in pain from pre-treatment to post-treatment (Surgical treatment effect): 0.02 (SD = 0.4; 95% CI = -0.8 to 0.8; p = 0.96); Post-treatment to discharge (Surgical treatment effect): 1.85 (SD = 0.6; 95% CI = -0.7 to 3.0; p = 0.002); Last 24 hours of admission (Surgical treatment effect): 1.2 (SD = 0.5; 95% CI = 0.2 to 2.2; p = 0.021); 48 hours into admission (Surgical treatment effect): -0.15 (SD = 0.5; 95% CI = -1.1 to 0.8; p = 0.764); Narcotic use (mg) – 48 hours into admission (Surgical treatment effect): 3.9 (SD = 16.1; 95% CI = -28.1 to 35.9; p = 0.811); Last 24 hours of admission (Surgical treatment effect): -0.49 (SD = 8.7; 95% CI = -17.7 to 16.7; p = 0.955)	Days-to-mobilisation - Surgical treatment effect: -1.7 (SD 0.08; 95% CI -3.3 to -0.01; p=0.034)	Not reported
Höch et al. [17}	EQ-5D - Surgical: 91.8 (SD = 9.3), non-surgical: 89.5 (SD = 18.4); SF-36 - entire cohort PCS Mean: 44.8 (SD 10), entire cohort MCS Mean: 52.6 (SD = 15) (no significant difference between surgical/non-surgical cohorts)	10-point VAS - Surgical: 2.6 (SD = 2.1), non-surgical: 2.8 (SD = 2.5)	Overall complication rate (%) - Surgical: 6 (SD = 16.7), non-surgical: 4 (SD = 11.5; p > 0.5); Infection to anterior plate – n = 1; injury to bladder - Surgical: n = 1, non-surgical: n = 1	Surgical: Mean = 14 days (range = 1–71), non-surgical: Mean = 13 days (range = 3–35; p > 0.05)
Petryla et al. [16]	Majeed score - Change in score with surgical management: -34.08 (SD = 18.95), change in score with non-surgical management: -31.44 (SD = 14.41; p = 0.542); SF-36 - Change in PCS with surgical management: -19.45 (SD = 9.95), Change in PCS with non-surgical management: -19.36 (SD = 7.88; p = 0.687), change in MCS with surgical management: -6.38 (SD = 11.04), Change in MCS with non-surgical management: -7.23 (SD = 10.86, p = 0.816)	Bodily pain score (of SF-36) - Change in score with surgical management: -47.3 (SD = 29.02), Change in score with non-surgical management: -40.72 (SD = 25.58; p = 0.445)	Surgical complications - Complication rate: 18.9%, wound infection (n = 1), screw migration (n = 2), S1 neuropathy (n = 4)	Not reported
Tornetta et al. [19]	Not measured	10-point VAS - 6 weeks post-surgical: 3.5, 6 weeks post non-surgical: 5.2 (p = 0.033); 12 weeks post-surgical: 2.9, 12 weeks post non-surgical: 4 (p = 0.019)	Not measured	Not reported
Zwingmann et al. [18]	EQ-5D - Surgical: 0.88 (SD = 0.14), non-surgical: 0.85 (SD = 0.14; p = 0.965); Merle d’Aubigne score - surgical: 15.9 (SD = 2.2), non-surgical: 16.3 (SD = 2.2; p = 0.768)	10-point VAS - Surgical: 7.1 (SD = 2.0), non-surgical: 7.7 (SD = 2.1, p = 0.508)	Not measured	Surgical: Mean = 16 days (SD = 7.3), non-surgical: Mean = 12.5 days (SD = 7.4; p = 0.315)

Risk of Bias

Two studies had a serious risk of bias due to confounding, while the remaining three had a moderate risk of bias, according to the ROBINS-I tool. Appendix 2 contains the detailed risk of bias assessments for the included studies.

Primary Outcome Analysis

Patient-reported outcome measures: quality of life (QoL): PROMs were measured in three studies; two studies used the EQ-5D-3L questionnaire [18,19], two employed the SF-36 [17,18], and two reported the Majeed score and Merle d’Aubigne hip score [17,19].

The EQ-5D questionnaire is a standardised scoring system that assesses a patient’s QoL across five domains, including physical and mental health (scale: -0.594 to 1.000, where 0 represents death, and less than 0 represents a QoL worse than death). After a follow-up of at least one year, Höch et al. observed no significant difference in EQ-5D between surgical (mean: 91.8, SD: 9.3) and non-surgical (mean: 89.5, SD: 18.4; p > 0.05) treatment groups (EQ-5D scores were multiplied by 100). Similarly, after a mean follow-up of 4.3 years, Zwingmann et al. reported no significant differences in EQ-5D scores between the surgical (mean: 0.88, SD: 0.14) and non-surgical groups (mean: 0.85, SD: 0.14; p = 0.965).

The SF-36 questionnaire (scale: 0-100, where the lower limit represents extreme disability/symptoms, and the upper limit represents no disability/symptoms) is another standardised scoring system that assesses a patient’s QoL. Petryla et al. measured the SF-36 scores of the respective cohorts at two time points (pre-injury and 10 weeks post-injury) and found no statistically significant differences in the change of the mental component summary (MCS) (surgical mean: -6.38, SD: 11.04; non-surgical mean: -7.23, SD: 10.86; p = 0.816) or physical component summary (PCS) (surgical mean: -19.45, SD: 9.95; non-surgical mean: -19.36, SD: 7.88; p = 0.687). Höch et al. also evaluated the SF-36 at least one year post-injury, and there was no statistically significant difference in mean MCS and PCS SF-36 scores (p > 0.05) between treatment groups.

Patient-reported outcome measures: function: Zwingmann et al. used the Merle d’Aubigne hip score (scale of 0-6 for each domain of walking, pain, and mobility, where 6 represents the best condition and 0 represents the worst condition), and Petryla et al. used the Majeed score (a standardised survey to assess functional outcomes after pelvic fractures, with domains focused on mobility, sexual intercourse, and pain, scored from 0 to 100, where <55 represents poor function, and ≥85 represents excellent function) to assess functional status. Zwingmann et al. discovered no statistically significant differences in functional status between treatment groups after a mean 4.3-year follow-up (surgical mean: 15.9, SD: 2.2; non-surgical mean: 16.3, SD: 2.2; p = 0.768). Similarly, when assessed 10 weeks post-injury by Petryla et al., the Majeed score showed no statistical difference between cohorts (surgical mean change: -34.08, SD: 18.95; non-surgical mean change: -31.44, SD: 14.41; p = 0.542).

Pain: Four studies assessed pain using the VAS (scale: 0-10, where 0 represents no pain, and 10 represents the worst pain) [15,16,18,19]. Some results were presented as an overall surgical treatment effect, a single figure denoting the difference between the mean score in the non-surgical cohort versus the surgical cohort. The surgical cohort in Hagen et al. had a statistically significant improvement in VAS pain scores from post-treatment to discharge from the hospital (surgical treatment effect [non-surgery score minus surgery score]: 1.85, SD: 0.6, 95% CI: -0.7 to 3.0; p = 0.002) and in the last 24 hours of admission (surgical treatment effect: 1.2, SD: 0.5, 95% CI: 0.2 to 2.2; p = 0.021) compared to the non-surgical cohort. In addition to the VAS, Hagen et al. also measured narcotic usage 48 hours into the admission (surgical treatment effect: 3.9, SD: 16.1, 95% CI: -28.1 to 35.9; p = 0.811) and in the last 24 hours of admission (surgical treatment effect: -0.49, SD: 8.7, 95% CI: -17.7 to 16.7; p=0.955) but these results yielded no statistical significance.

Tornetta et al. observed that the surgical cohort had better VAS scores six weeks post-injury (surgical mean: 3.5; non-surgical mean: 4; p = 0.033) and 12 weeks post-injury (surgical mean: 2.9; non-surgical mean: 4; p = 0.019). Conversely, in the two studies where patients were followed up over a longer period, the changes in VAS score were negligible; this was noted in Zwingmann et al. (surgical: 7.1, SD: 2.0; non-surgical: 7.7, SD: 2.1; p = 0.508) and Höch et al. (surgical: 2.6, SD: 2.1; non-surgical: 2.8, SD: 2.5; p > 0.05) after a mean follow-up of 52 months and 47 months, respectively.

Secondary Outcome Analysis

Length of hospital stay: Two studies reported the length of hospital stay [17,18]. Höch et al. reported a mean length of 14 days (range: 1-71) for the surgical cohort and 13 days (range: 3-35) for the non-surgical cohort, with no statistical difference (p > 0.05). Zwingmann et al. reported no significant difference between treatment groups (surgical mean: 16 days, SD: 7.3; non-surgical mean: 12.5 days, SD: 7.4; p = 0.315).

Associated complications: Complications after treatment of LC1 injuries were discussed in two studies [16,17]. Höch et al. were the only researchers to detail complication rates in both treatment groups. The surgical cohort had a complication rate of 6% versus 4% in the non-surgical cohort, with no statistical significance (p > 0.05). Bladder injury, pneumonia, acute respiratory distress syndrome, surgical malpositioning, and infected metalwork were among the reported complications. Petryla et al. only reported the complications in the surgical cohort, including wound infection (n = 1), screw migration (n = 2) and S1 neuropathy (n = 4), yielding a total surgical complication rate of 18.9%.

Days to mobilisation: Hagen et al. measured the number of days required for the patient to transfer independently from the bed to the chair. The surgical treatment group had a mean reduction of 1.7 days to independent mobilisation compared to the non-surgical group (mean difference (surgical minus non-surgical): -1.7 days, SD: 0.08, 95% CI: -3.3 to -0.01; p = 0.034).

Discussion

This is the first systematic review to investigate the surgical versus non-surgical treatment of unstable LC1 injuries in the non-fragility fracture population. Five observational studies were eligible for inclusion in this study. Two of the included studies displayed a serious risk of bias, with the remainder deemed to display moderate risk.

Three studies used the SF-36 or EQ-5D to assess QoL, with no statistical differences between treatment groups. Two studies also demonstrated no correlations between functional outcomes at a mean follow-up of ten weeks and four months, respectively.

Surgical fixation was positively correlated with reduced pain in the short term, particularly from the postoperative period to discharge from the hospital and up to 12 weeks post-injury. Surgical fixation was also found to reduce the time to mobilisation by 1.7 days, measured in one study. However, in two studies investigating longer-term outcomes, there was no statistical difference in pain between treatment groups after four to five years. Despite these results, there is ongoing debate regarding the minimal clinically important difference (MCID) for VAS in patients with these injuries. The VAS MCID for pain due to osteoarthritis of the knee, for example, has been demonstrated to be a 2.5 score reduction for surgical or rehabilitative therapy [20]. In the aforementioned study, the differences in VAS were 1.7 and 1.1 at six weeks and 12 weeks follow-up, respectively. Nonetheless, the VAS has proven reliable for pain measurement among patients of all ages and educational levels [21].

Few studies investigated post-treatment complications but found no statistical difference in their results, albeit they are likely to be underpowered for determining true statistical significance. In Höch et al., although the overall complication rate was negligible between the non-surgical and surgical cohorts (11.5 vs. 16.7%, p > 0.5), 8% of the complications in the surgical group were directly related to the surgery.

Although LC1 injuries include both unstable and stable fractures, this review has focused on those with unstable injury patterns. Currently, there are conflicting views on what constitutes an unstable LC1 injury, with some authors basing their criteria on the degree of fracture displacement [22] and others on the patient’s pain, for example [12]. A recent cadaveric study also revealed that the displacement of LC1 injuries may be related to pubic rami fracture morphology with oblique pubic rami fractures being most susceptible to displacement over time [23].

Treatment of LC1 injuries varies with no agreed algorithm for determining whether patients would be better off receiving surgical or non-surgical care [24]. Beckmann et al. used the expert opinion of 111 orthopaedic surgeons to develop a validated radiographic scoring system for LC1 fractures [24]. Specific parameters, such as ramus displacement, Denis classification, and sacral displacement, were included in the system, with a score greater than nine out of 14 indicating the need for surgery [25]. Similar efforts have been proposed by Sagi et al. suggesting >1 cm and Giannoudis et al. advising ≥2 cm of pubic rami displacement as cut-offs for surgical intervention [8,26]. Despite these efforts, Hadeed et al. outlined that fellowship-trained orthopaedic surgeons often exhibit inconsistent measurement of sacral fracture completeness in LC1 injuries [27].

Although there have been attempts to study the optimum management algorithm, only one clinical trial has been published to date. In patients with minimally displaced LC fractures of the pelvis, Slobogean et al. discovered that surgical fixation results in a minor but sustained improvement in pain and function for up to 12 months. Patients with a 5 mm displacement were the best surgical candidates, with the most short-term pain relief after surgery [28]. However, because the cohort contained both LC1 and LC2 fractures, this study was excluded from our review.

Lykomitros et al. demonstrated that patients with sacral fractures treated non-surgically had better SF-36 scores in all domains than those treated surgically. The authors highlighted that patients treated surgically had more concomitant injuries, resulting in a higher Injury Severity Score (ISS), a possible confounder for the lack of improvement in QoL following surgical management [29]. Similarly, Tosounidis et al. reported that surgical stabilisation of LC1 injuries reduced pain and analgesic requirements significantly in the immediate post-injury period [26]. However, this study was excluded from our review due to the absence of complete sacral fractures in the non-surgical comparison group.

The existing literature is very limited in the reporting of function and postoperative complication outcomes. A feasibility study has been conducted [30], demonstrating that it would be feasible to recruit into a definitive RCT comparing the surgical and non-surgical treatment of LC1 fractures. A trial could further investigate PROMs, function, pain, QoL, complications, and economic evaluations.

Our review has several limitations. All of the included studies were observational, inherently increasing the risk of confounding and selection bias; this was emphasised in the risk of bias assessment, as all studies were of moderate-to-serious risk of bias. The severity of concomitant injuries was not controlled for; for example, a polytraumatised patient may be more likely to undergo surgical intervention than a patient with an isolated unstable LC1 fracture. The surgical group tended to have more severe injuries, perhaps more significant pain, and reduced QoL. We were unable to perform a formal meta-analysis due to heterogeneity of the time points at which outcomes were measured in individual studies and a lack of consistency in reporting. There were variations in surgical technique, which further increased the risk of confounding. Furthermore, there is no widely accepted protocol for LC1 fracture management, and each study had variable eligibility criteria for surgical intervention: some placing greater emphasis on pain and mobility, and others on initial displacement on static radiographs to aid decision-making.

## Conclusions

Unstable LC1 fractures are a heterogenous injury pattern, with no current agreed protocol for surgical intervention. The studies that met our eligibility criteria demonstrated small improvements in short-term pain and days-to-mobilisation with surgical fixation, but no statistically significant differences in functional outcomes or QoL; however, all included studies were of moderate-to-serious risk of bias. This review highlights the low quantity and quality of existing data on patients with unstable LC1 pelvic non-fragility fractures and the need for a definitive RCT to determine whether surgical or non-surgical care should be the preferred treatment in terms of clinical and cost-effective care.

## Figures and Tables

**Figure 2:**
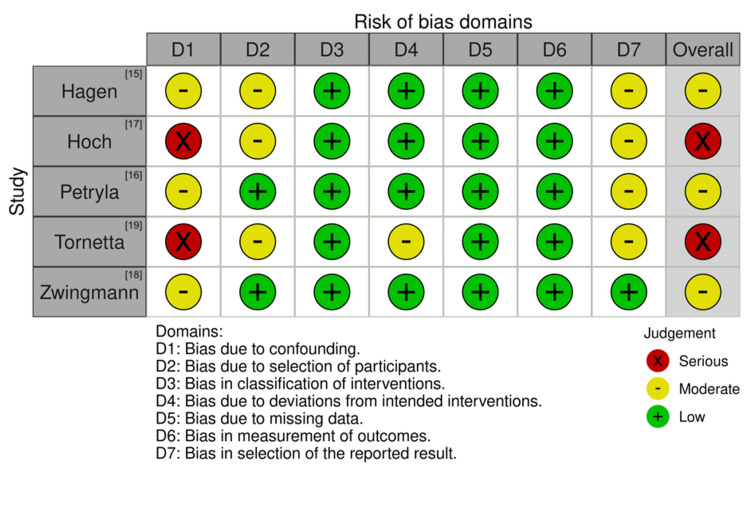
Risk of bias scoring. [15-19].

**Figure 3:**
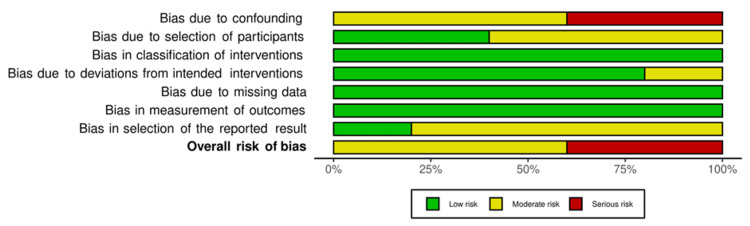
Risk of bias scoring.

## Appendices

Appendix 1: Detailed search strategy

Search strategy for Ovid MEDLINE(R) ALL

From inception to 21 June 2021

1          Fractures, Compression (2538)

2         Pelvic Bones (9923)

3         1 and 2 (47)

4         “LC1” or “LC-1” mp. (806)

5         “lateral compression” adj3 fractur$ .mp. (71)

6         “pelvic ring” adj3 fractur$ .mp. (791)

7         “lateral compression” adj3 injur$ .mp. (77)

8         or/4-7 (1689)

9         3 or 8 (1711)

Search strategy for Embase

From inception to 21 June 2021

1          compression fracture/ (6665)

2         pelvis fracture/ (7236)

3         1 and 2 (137)

4         “LC1” or “LC-1” .mp. (1092)

5         “lateral compression” adj3 fractur$ .mp. (86)

6         “pelvic ring” adj3 fractur$ .mp. (913)

7         “lateral compression” adj3 injur$ .mp. (85)

8         or/4-7 (2111)

9         3 or 8 (2204)

Search strategy for Cochrane Central

From inception to 21 June 2021

#1        MeSH descriptor: [Fractures, Compression] explode all trees (154)

#2       MeSH descriptor: [Pelvis] explode all trees (978)

#3       #1 and #2 (0)

#4       “LC1” or “LC-1” (50)

#5       “lateral compression” (20)

#6       “pelvic” or “pelvis” (17472)

#7       (#4 or #5) AND 6 (10)

Search strategy for Ovid Medline(R) All

From inception to 05 June 2022

1          Fractures, Compression (2883)

2         Pelvic Bones/ (10359)

3         1 and 2 (56)

4         “LC1” or “LC-1” mp. (863)

5         “lateral compression” adj3 fractur$ .mp. (79)

6         “pelvic ring” adj3 fractur$ .mp. (859)

7         “lateral compression” adj3 injur$ .mp. (86)

8         or/4-7 (1825)

9         3 or 8 (1849)

10        limit 9 to last year (229)

Search strategy for Embase

From inception to 05 June 2022

1          compression fracture/ (7245)

2         pelvis fracture/ (7750)

3         1 and 2 (147)

4         “LC1” or “LC-1” .mp. (1169)

5         “lateral compression” adj3 fractur$ .mp. (94)

6         “pelvic ring” adj3 fractur$ .mp. (1003)

7         “lateral compression” adj3 injur$ .mp. (90)

8         or/4-7 (2283)

9         3 or 8 (2384)

10        limit 9 to last year (275)

Search strategy for Cochrane Central

From inception to 05 June 2022

#1        MeSH descriptor: [Fractures, Compression] explode all trees (170)

#2       MeSH descriptor: [Pelvis] explode all trees (1080)

#3       #1 and #2 (2)

#4       “LC1” or “LC-1” (52)

#5       “lateral compression” (24)

#6       “pelvic” or “pelvis” (18759)

#7        (#4 or #5) AND 6 (13)

Appendix 2: Risk of bias scores
